# 

**Published:** 1903-01-24

**Authors:** 


					'mhe hospital. "The standard of highest purity."?Lancet. Jan. 24th, 1903.
CADBURY'SS ^ mjr ?. CADBURY'S
OOGOA
ABSOLUTELY PURE. ^ ^ NO DRUGS OR *LKaLiB8 UUD.
OOOOA
tsoati Wes term hiFutuksx
No, 852. Vol. XXXIII. [KSS?]
Saturday, Jan. 24th, 1903.
[SnhscriptbnTerms,] pRICH TWOPENCE.

				

